# Etiologic profile and antimicrobial susceptibility of community-acquired urinary tract infection in two Cameroonian towns

**DOI:** 10.1186/1756-0500-5-219

**Published:** 2012-05-07

**Authors:** Jane-Francis Tatah Kihla Akoachere, Suylika Yvonne, Njom Henry Akum, Esemu Nkie Seraphine

**Affiliations:** 1 Department of Microbiology and Parasitology, Faculty of Science, University of Buea, Buea, Cameroon; 2 Laboratory for Emerging Infectious Diseases, Faculty of Science, University of Buea, Buea, Cameroon

**Keywords:** Community-acquired urinary tract infection, Bacteria, Antibiotic susceptibility, Cameroon

## Abstract

**Background:**

Urinary tract infection (UTI) represents one of the most common diseases encountered in community medical practice. In resource poor settings, treatment is usually empiric due to the high cost and long duration required for reporting diagnosis by culture and antibiotic susceptibility testing. With the growing problem of drug resistance knowledge of antibiotic susceptibility pattern is pertinent for successful eradication of invading pathogens. Our study, the first of its kind in Cameroon, analyzed the distribution and antibiotic susceptibility of bacteria causing community-acquired urinary tract infection (CAUTI) in two towns (Bamenda and Buea) with a large number of young and middle aged persons, to provide data that could guide empiric treatment.

**Findings:**

We cultured 235 urine specimens and analyzed the antibiotic susceptibility of isolates by the disc diffusion technique. Uropathogens were recovered from 137 (58.3%), with prevalence rates in Buea and Bamenda being 65.9% and 54% respectively. Predominant pathogens were *Escherichia coli* (31.4%), *Klebsiella oxytoca* (25.5%) and *Staphylococcus spp* (24.1%). Geographic variation in uropathogen distribution and antibiotic susceptibility was observed, and a significant difference in pathogen distribution with respect to gender. The 20–39 years age group had the highest prevalence of infection. All pathogens isolated were detected in this group. Isolates exhibited low susceptibility to antibiotics tested. Bamenda isolates generally exhibited lower susceptibility compared to those from Buea.

**Conclusion:**

Regional variation in etiology of CAUTI and antibiotic susceptibility observed in our study emphasizes the need to establish local and national antimicrobial resistance monitoring systems in Cameroon to provide information for the development of CAUTI treatment guidelines.

## Findings

A total of 235 patients comprising 150 (63.8%) from Bamenda and 85 (36.2%) from Buea were sampled. Of these, 167 (71.1%) were females and 68 (38.9%) were males. The age of participants ranged from 2 years to 80 years. Patients were stratified into the following age-groups: < 20 years (n = 29, 12.3%), 20–39 years (n = 131, 55.7%), 40–59 years (n = 51, 21.7%) and ≥ 60 years (n = 24, 10.2%). A total of 137 (58.3%) samples had significant growth of pathogens.

UTI prevalence was 65.9% in Buea and 54% in Bamenda (Table 1). Logistic regression analysis of UTI with study site as a predictor produced no significant relationship (G = 0.161, df = 1, *p* = 0.687). Measure of association between prevalence of UTI and study site showed weak predictive ability (Somers’ D (0.01) and Goodman-Kruskal Gamma (0.11) are close to zero).

**Table 1:** Prevalence of infection in study area with respect to gender

**Study Site (Region)**	**Gender**	**No. positive**	**% positive**
Bamenda (North West)	Females, n = 97	65	67.0%
	Males, n = 53	16	30.2%
**Total**	150	81	54.0%
Buea (South West)	Females, n = 70	48	68.6%
	Males , n =15	8	53.3%
**Total**	85	56	65.9%
**Grand total**	Females, n = 167	113	67.7%
	Males, n = 68	24	35.3%
	Males + females = 235	137	58.3%

Analyzing prevalence with respect to gender, females (67.7%) had a higher prevalence of infection than males (35.3%) (Table 1). UTI prevalence was significantly related to gender (G = 20.769, DF = 1, P-Value = 0.000) with projections of 1.346 higher prevalence in females relative to males. Large values of Somers’ D (0.27) and Goodman-Kruskal Gamma (0.59) were associated with this relationship indicating good predictive ability. A combined logistic regression using gender, study site versus UTI showed a significant relationship (G = 23.419, DF = 2, P-Value = 0.000) and good predictive power (Somers’ D (0.29), Goodman-Kruskal Gamma (0.58)).

**Figure 1:**
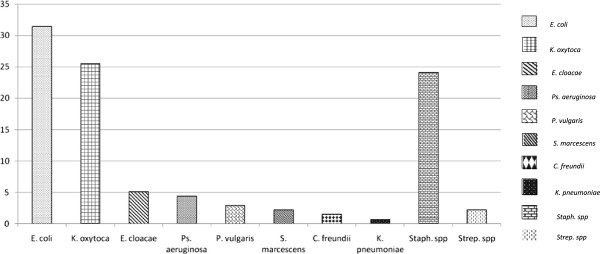
**Prevalence of bacterial isolates in samples.** Ten bacterial species were isolated. *E. coli, K. oxytoca* and *Staph. spp* were the predominant pathogens isolated while *K. pneumoniae* was the least.

*E. coli* (31.4%), *K. oxytoca* (25.5%) and *Staph*. sp (24.1%) were the predominant isolates associated with UTI while *K. pneumoniae* had the least occurrence (1/81, 1.2%) (Figure 1).

**Figure 2:**
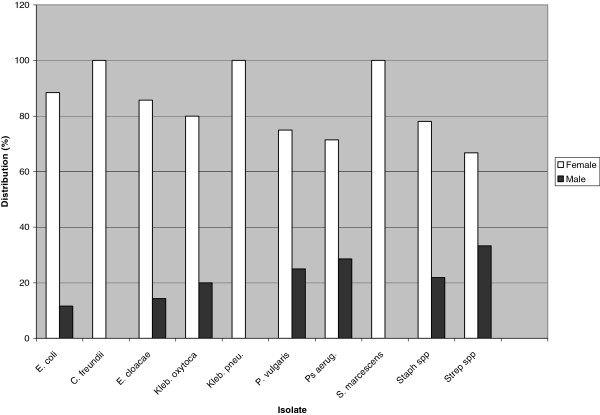
**Distribution of uropathogens with respect to gender.** Prevalence of pathogens was higher in females than in males. All isolates from males were detected in females. *C. fruendii, Klebsiella pneumoniae* and *Serratia marcescens* were isolated only from female participants.

Gram-positive organisms constituted only 26.3% of isolates (Table 2). *Klebsiella oxytoca* was the most frequently isolated organism in Bamenda (29.6%). This was closely followed by *E. coli* (28.4%) and *Staph*. spp (22.2%). The predominant pathogen in Buea was *E. coli* (35.7%). *Streptococcus* spp. (3/56, 5.4%) were isolated only from samples from Buea while *Proteus vulgaris* (4/81, 4.9%) and *K. pneumoniae* (1/81, 1.2%) were detected only in samples from Bamenda. There was no significant relationship between uropathogen distribution and study site (G = 1.665, DF = 1, P-Value = 0.197). *Citrobacter freundii, K. pneumoniae* and *Serratia marcescens* were isolated only from female participants (Figure 2). Predominant pathogens in females were *E. coli* (88.4%), *Enterobacter cloacae* (85.7%) and *K. oxytoca* (80%) (Figure 2). In males the most frequently isolated organisms were *Strep*. spp (33.3%), *Pseudomonas aeruginosa* (28.6%) and *P. vulgaris* (25.0%). All bacteria isolated from males were also isolated from females.

**Table 2:** Distribution of uropathogens in study sites

**Isolate**	**Bamenda (%)**	**Buea(%)**	**Total (%)**	**Gram reaction**
*E. coli*	23 (28.4 )*	20 (35.7 )*	43 (31.4 )	Gram-negative n = 101 (73. 7%)
*K. oxytoca*	24 (29.6)	11 (19.6)	35 (25.5)	
*E. cloacae*	5 (6.2)	2 (1.8)	7 (5.1)	
*Ps. aeruginosa*	3 (3.7)	3 (5.4)	6 (4.4)	
*P. vulgaris*	4 (4.9)	0 (0.0)**	4 (2.9)	
*S. marcescens*	2 (2.5)	1 (1.8)	3 (2.2)	
*C. freundii*	1 (1.2)	1 (1.8)	2 (1.5)	
*K. pneumoniae*	1 (1.2)	0 (0.0)**	1 (0.7)	
*Staph.* spp	18 (22.2)	15 (26.8)	33 (24.1)	Gram-positive n = 36 (26.3%)
*Strep. s*pp	0 (0.0)**	3 (5.4)	3 (2.2)	
**Total**	**81 (59.1)**	**56 (40.9)**	**137 (100)**	

*Most prevalent isolate in study site; **Isolated not recovered from study site.

*E. coli, E. cloacae, K. oxytoca* and *Staph.* spp were isolated from all age-groups (Table 3). All isolates were detected in the 20–39 years group. With the exception of the 40–59 years group in which the predominant isolate was *K. oxytoca* (40.7%), *E. coli* was the predominant isolate in all the other age groups. In addition to *E. coli* (26.7%), other predominant isolates in the < 20 years participants were *K. oxytoca* (26.7%) and *Staph.* spp (26.7%). *K. pneumoniae* (1.2%) was isolated solely from 20–39 years age-group. Strata of age gave insufficient evidence for a significant relationship with uropathogens (G = 0.824, DF = 3, P-Value = 0.844). However, the logistic regression coefficient of age strata; 0.040,–0.296, 0.038 for 20–39, 40–59, 60+ respectively showed an increasing uropathogens distribution order of 60 + < (< 20) < (40–59) < (20–39). Both the Pearson and Deviance tests had p-values (0.920 and 0.852 respectively) greater than 0.05 indicating that there was insufficient evidence for the model not fitting the data adequately.

**Table 3:** Distribution of isolates with respect to age of participants

**Isolate**	**Age-group**				
	**< 20 (%)**	**20–39 (%)**	**40–59 (%)**	**≥ 60 (%)**	**Total (%)**
*E. coli*	4 (26.7)*	29 (35.8)*	5 (18.5)	5 (35.7)*	43 (31.4)
*K. oxytoca*	4 (26.7)*	17 (21)	11 (40.7)*	3 (21.4)	35 (25.5)
*Staph.* spp	4 (26.7)*	18 (22.2)	6 (22.2)	4 (28.6)	33 (24.1)
*E. cloacae*	1 (6.7)	4 (4.9)	1 (3.7)	1 (7.1)	7 (5.1)
*Ps. aeruginosa*	0**	3 (3.7)	2 (7.4)	1 (7.1)	6 (4.4)
*P. vulgaris*	0**	3 (3.7)	1 (3.7)	0**	4 (2.9)
*S. marcescens*	0**	2 (2.5)	1 (3.7)	0**	3 (2.2)
*Strep. S*pp	1 (6.7)	2 (2.5)	0**	0**	3 (2.2)
*C. freundii*	1 (6.7)	1 (1.2)	0**	0**	2 (1.5)
*K. pneumoniae*	0**	1 (1.2)	0**	0**	1 (0.7)
**Total**	**15 (10.9)**	**81 (59.1)**	**27 (19.7)**	**14 (10.2)**	**137 (100)**

*Most prevalent pathogen in age group; **Pathogen not detected in age group.

### Antimicrobial susceptibility of isolates

The antimicrobial potency and spectrum of 8 classes of antibiotics against uropathogens is summarized in Table 4. Cotrimoxazole was the most inactive drug as isolates showed very low susceptibilities: 12.5% for Buea and 1.9% for Bamenda. However, all (100%) *E. coli* isolates from Buea were sensitive to the drug. Isolates were also resistant to ampicillin and nitrofurantoin. *E. coli* isolates from Bamenda were most sensitive to ceftriaxone (65%) (Table 4). All *K. oxytoca* isolates from Buea were sensitive to ciprofloxacin (100%) and gentamicin (100%) whereas the most potent drugs against the Bamenda isolates were gentamicin (91.2%), ciprofloxacin (87.5%) and nitrofurantoin (83.3%). Isolates generally exhibited susceptibility greater than 50% to ciprofloxacin except for *S. marcescens, Strep. spp* and *E. cloacae* from Buea, and *C. fruendii* from Bamenda which were all (100%) resistant. Overall, isolates from Buea showed highest susceptibility to gentamicin (80.9%) whereas ciprofloxacin (65.0%) was the most active agent against Bamenda isolates. With a few exceptions, Bamenda isolates generally exhibited lower susceptibilities than Buea isolates.

**Table 4:** Antibiotic susceptibility of isolates from each region (%)

**ISOLATE**	**AMP** **BU BD**		**CIP** **BU BD**		**COT** **BU BD**		**ERY** **BU BD**		**GEN** **BU BD**		**NIT** **BU BD**		**CEF** **BU BD**	
*E. coli*	93.3	26.1	100	52.2	100	17.4	100	39.1	80	43.5	20	43.5	35	65
*K. oxytoca*	36.4	36.4	100	87.5	0	0	0	29.2	100	91.2	90.9	83.3	18.2	75
*Staph. sp*	40	77.7	80	55.6	0	0	33.3	55.6	66.7	55.6	40	66.7	53.3	72.2
*E. cloacae*	100	0	0	80	0	0	100	50	100	80	0	80	100	100
*P. aeruginosa*	0	66.7	100	100	0	0	0	0	100	100	100	66.7	66.7	100
*P. vulgaris*	ND	0	ND	50	ND	0	ND	50	ND	25	ND	50	ND	25
*S. marcescens*	0	50	0	100	0	0	100	0	100	100	0	50	0	50
*Strept. spp*	100	ND	0	ND	0	ND	100	ND	100	ND	0	ND	100	ND
*C. fruendii*	0	0	100	0	0	0	0	100	0	100	0	0	0	0
*K. pneumoniae*	ND	0	ND	100	ND	0	ND	0	ND	0	ND	0	ND	0
**Mean %**	**46.2**	**28.5**	**60**	**65.0**	**12.5**	**1.9**	**54.2**	**36.0**	**80.8**	**55.1**	**31.4**	**48.9**	**46.7**	**54.1**

**BU**- Buea; **BD**-Bamenda; **ND**-Not determined as isolate was not recovered from study site; **AMP**-Ampicillin; **CIP**-Ciprofloxacin; **COT**-Cotrimoxazole; **ERY**-Erythromycin; **GEN**- Gentamicin; **NIT**- Nitrofurantoin; **CEF**- Ceftriaxone.

## Background

Urinary tract infection (UTI) is the second most common infectious presentation in community medical practice. Worldwide, about 150 million people are diagnosed with UTI each year costing the global economy in excess of 6 billion dollars [1]. The incidence of these infections among young sexually active women has been reported to exceed 0.5 episodes per year [2] with 20 to 30% of women experiencing recurring infections. During reproductive life this infection represents a great amount of work disabilities, hence the need for prophylaxis and prompt treatment. Most of the UTI seen in women are uncomplicated (occurring in otherwise healthy individuals without any metabolic, functional or anatomic abnormality of the urinary tract). However, after middle age, the prevalence and incidence of UTI increase progressively in men with a concomitant and progressive decrease in the male-to-female ratio [3]. Major contributing factors for high levels of UTIs particularly in rural areas are poor sanitary conditions and lack of proper hygiene.

Reporting of antimicrobial susceptibility testing of the urinary tract is usually achieved 48 h following sampling, and therefore, in the majority of community-acquired UTI (CAUTI), treatment decision is empiric, based on the limited and predictable spectrum of etiological microorganisms and available data reflecting antibiotic resistance. Furthermore, in most cases, culture and susceptibility testing costs more than antibiotic treatment itself. These factors have complicated empiric treatment of CAUTI as data on uropathogens prevalence and antimicrobial susceptibility becomes more difficult to obtain. Considering the fact that as with many community-acquired infections, resistance rates to antimicrobials commonly used in treatment of UTI is increasing and susceptibility of microorganisms shows significant geographical variations [4], studies to increase knowledge on etiologic agents of UTIs and their resistance patterns to antibiotics at the local and national levels are very important to guide clinicians in empiric treatment.

The etiology and resistance pattern of community-acquired uropathogens has not been extensively studied in Cameroon. Due to the high cost associated with diagnosis of UTI by culture, laboratory diagnosis of UTI in most health care facilities is mainly by urinalysis and urine microscopy. Although the etiology of UTI is predictable, determination of antimicrobial susceptibility pattern of pathogens is unattainable with these techniques. Therefore treatment of UTI is mainly empiric, without knowledge of antibiotic susceptibility of invading pathogen. With the growing problem of drug resistance in Cameroon as demonstrated by recent studies [5,6] there is an urgent need for continuous surveillance of antibiotic susceptibility of uropathogens. Appropriate knowledge of local and national antimicrobial resistance trends is of utmost importance in order to step up evidence based recommendations in empiric antibiotic treatment of UTI. Data on etiology of UTI as well as local and national antimicrobial susceptibility of uropathogens in Cameroon is scarce. This study was aimed at analyzing the distribution of species of bacterial pathogens associated with community-acquired UTI in patients in two Cameroonian towns: Bamenda (North West region) and Buea (South West region) and to determine their susceptibility to commonly prescribed antibiotics so as to generate data that could be helpful in improving the efficacy of empiric treatment of UTI. These towns were selected because of their location in distinct geographic regions and also because they host many educational institutions and as such have a large number of young and middle age individuals. Findings would also be helpful when formulating policies on antibiotic prescription.

## Methods

### Study area

The study was carried out in Bamenda and Buea. Bamenda is the capital city of North West (NW) region and has a population of over 269,530. It is characterized by high relief, cool temperatures (particularly in the dry season), heavy rainfall and savanna vegetation. Buea, the capital of the South West (SW) region is located on the eastern slopes of Mount Cameroon and has a population of over 200,000. Because of its location at the foot of Mount Cameroon, it has tropical and mountain rainforest as well as savanna vegetation. The climate is humid. Extended periods of rainfall, characterized by incessant drizzle and damp fogs are common during the rainy season. Buea and Bamenda are located 222 km apart.

### Study design, data collection and patients

This study was based on laboratory investigations with urine samples collected from all hospitalized and outpatients (those for which urinalysis was requested) in selected hospitals in Bamenda and Buea between 01 March, 2009 and 28 February, 2010. Participating hospitals were chosen due good patient turn out. The study included all patients having clinical evidence of UTI with symptoms less than 7 days presenting at the out-patient department of hospitals and those admitted for less than 48 h. All patients sampled gave consent to participate in the study. Patients with pregnancy, complicating factors such as upper UTI, urinary abnormalities or more than three episodes of UTI in the previous year were excluded. Also excluded from this study were patients with clinical symptoms of UTI but whose samples showed no growth and those who underwent antibiotic treatment within 48 h. Patients’ demographic data, symptoms and physical examination were collected using a questionnaire. Only one sample was collected from each patient. Ethical approval of the study was obtained from participating hospitals (Bamenda Regional Hospital, St. Mary Health Center Bamenda, Regional Hospital Annex Buea and Mount Mary Health Center Buea) and from the Northwest and Southwest Regional Delegations of Public Health.

### Sample collection and analysis

Fifty milliliters of clean-catch midstream urine specimen was collected in a sterile Dynarex specimen container. Twenty-five milliliters was used in this study and the rest of the sample was used by the hospital laboratory for urinalysis. The semi-quantitative technique to determine significant bacteriuria was employed by using 0.01 mL calibrated wire loop to inoculate 5% blood agar and Eosine-Methylene Blue (EMB) agar with uncentrifuged urine. Culture plates were incubated at 37°C for 18–24 h. After inoculation of media, the remaining sample was centrifuged at 2000 rpm for 5 minutes and sediment used for microscopy. A specimen was considered positive if a single organism was isolated at a concentration of greater than10^5^ CFU/mL and associated with microscopy findings of greater than 10 leucocytes per high power field. Bacteria were identified by Gram’s stain and standard biochemical procedures. Identity of enterobacteriaceae was confirmed using the API 20E kit.

### Antibiotic susceptibility testing

Susceptibility of isolates to antimicrobial agents of different classes was assessed by the disk diffusion technique on Mueller-Hinton agar as described by the National Committee for Clinical Laboratory Standards (presently called Clinical Laboratory Standard Institute) [7]. The following antibiotics used for empiric treatment were analyzed: erythromycin (15 μg/mL), gentamicin (10 μg/mL), trimethoprim/sulfamethoxazole (2.5 μg/mL), nitrofurantoin (50 μg/mL), ampicillin (25 μg/mL), ceftriazone (10 μg/mL), ciprofloxacin (5 μg/mL) and pefloxacin (10 μg/mL) (Oxoid, Basingstoke, England).

### Statistical analysis

Data from study was inserted into two separate MS Excel spreadsheets that were later on merged to ensure correct data entry, coded and imported into Minitab 16 for statistical analysis. Logistic regression was used to test the relationship between predictors (variables in the study) and urinary tract infections (UTI) using p-values of the G statistics at an α-level of 0.05. Coefficients of factor levels were used for comparative analysis relative to reference factors. Goodness-of-fit statistics was used to compare the fits of different models. Measure of association was used between predicted probabilities and the response variable to test the predictive ability of the model. Descriptive statistics was employed to weigh the outcome of variables.

## Discussion

Urinary tract infections (UTIs) represent one of the most common diseases encountered in medical practice, causing significant associated morbidity and occurring from neonate to the elderly [8]. Studies [9] have demonstrated geographic variation in etiologic characteristics of UTI and their resistance patterns to antibiotics. Therefore to successfully eradicate UTI by empiric treatment, knowledge of local etiologic agents and their antibiotic susceptibility is of great value. This study reports the etiologic agents of CAUTI and their antibiotic susceptibility in two Cameroonian cities located 222 km apart in two distinct geographic regions, and provides baseline data which could help in the establishment of local guidelines for treatment of CAUTI. Patients sampled were from the communities where the participating hospitals serve as main primary care centers.

Of the 235 suspected cases of UTI that were positive by microscopy (greater than 10 leucocytes per high power microscope field), only 137 (58.3%) were positive by culture (Table 1). Although the presence of white blood cells in urine signifies inflammation, based on our results, they do not always indicate UTI. Our findings therefore indicate that urine culture is essential for definitive diagnosis of UTI. The prevalence rate in Buea (65.9%) was higher than 54.0% recorded in Bamenda. In a similar study in Buea, Assob *et al.*[10] analyzed 53 urine samples from patients with UTI and reported a prevalence rate of 49.06%. Logistic regression analysis showed no relationship between UTI prevalence and study site (G = 0.161, df = 1, *p* = 0.687). Values reported in our study are higher than 35.2% reported by Yuyun *et al.*[11] in Yaounde, Cameroon. Prevalence rates reported in Cameroon are higher than values reported in other developing countries such as Senegal, 8.45% [12], Cambodia 22.53% [13], Latin America 29.9% [14], Rwanda, 19.3% [15] and India 10.86% [16] and 9.17% [17]. Thus UTI could be a significant cause of morbidity in Cameroon. Although UTI ranks among the most common infections in developing countries we sampled only 235 participants. In addition to the fact that uncomplicated UTIs are generally self-limiting, patients are usually concerned about the social stigma associated with UTI as such prefer unorthodox treatment or self medication. These factors could explain our small sample size.

UTI prevalence was higher in females (67.7%) than in males (35.3%) (Table 1). The prevalence of UTI was significantly related to gender (G = 20.769, DF = 1, P-Value = 0.000). The highest occurrence was in the age group 20–39 years (81/137, 59.1%) followed by participants 40–59 years old (27/137, 19.7%) (Table 3). This is in agreement with previous studies [16] which have demonstrated a high prevalence of UTI in individuals less than 50 years of age. However, recent studies [18] have associated older age with higher morbidity. The age group 20–39 years is the most sexually active group of the population and their activities predispose them to UTI. Most women of child-bearing age fall within this group. Thus, during reproductive life, UTI could cause a great amount of disabilities. The propensity of young women to develop UTI has been explained on the basis of their anatomy (especially a short urethra) and certain behavioral factors [19].

Gram negative organisms (73.7%) (Table 2) were the main cause of UTI in our study sites with *E. coli* (31.4%) being the predominant pathogen while *K. pneumoniae* was the least (1.2%) (Figure 1). UTI etiology as well as the predominance of *E. coli* as a leading cause of CAUTI reported in our study has been well documented in reports from other parts of the world such as the ECO SENS report from Europe and Canada [20], and The Surveillance Network (TSN) study from the United States [21] as well as numerous reports from developing countries [22,23]. The low prevalence of *K. pneumoniae* in cases of CAUTI is consistent with previous studies [24]. Although *E. coli, K. oxytoca* and *Staph. spp* were the principal uropathogens in our study sites, there was a remarkable variation in distribution of other etiologic agents between study sites. *P. vulgaris* (4.9%) and *K. pnuemoniae* (1.2%) were isolated only from samples collected from Bamenda whereas *Strep. spp* (5.4%) was detected solely in Buea samples. Even though the prevalence of these isolates was low, our data suggests evolution in uropathogen etiology and emphasizes the need for periodic assessment of uropathogens and their antibiotic susceptibility pattern at local and national level to guarantee successful empiric treatment. Due to the large number of isolates obtained from this study, we were unable to characterize the Gram positive isolates to permit their identification to species level. This constitutes a limitation to this study. All uropathogens predominated in females than in males (Figure 2). In addition we observed variation in distribution of etiologic agents with gender and age (Figure 2, Table 3). *C. fruendii, K. pneumoniae* and *S. marcescens* were isolated solely from females. All isolates were recovered from the age group 20–39 years whereas in participants over 60 years old, only 50% (5/10) of species were detected. Strata of ages gave insufficient evidence for a significant relationship with uropathogens (G = 0.824, DF = 3, P-Value = 0.844). However, the logistic regression coefficient of age strata; 0.040, -0.296, 0.038 for 20–39, 40–59, 60+ respectively showed an increasing uropathogens distribution order of 60 + < (< 20) < (40–59) < (20–39).

Our data demonstrates low susceptibility to first line agents and regional variation in antibiotic susceptibility pattern. Generally, ampicillin, cotrimoxazole and nitrofurantoin were the most inactive drugs as they exhibited susceptibilities less than 50% in both study sites. These low susceptibilities limit their usefulness in the treatment of UTI. All isolates were resistant (100%) to cotrimoxazole except *E. coli* with susceptibilities of 100% and 17.4% respectively for the Buea and Bamenda isolates. Other studies on community-acquired UTI [16] have reported low susceptibility of *E. coli* to cotrimoxazole. Although the Infectious Disease Society of America (IDSA) guidelines consider cotrimoxazole for empiric treatment of UTI [25], the low susceptibility recorded in our study (12.5% and 1.9% respectively for Buea and Bamenda isolates) shows that cotrimoxazole is not appropriate for empiric treatment of UTI in study sites but could be a suitable agent for eradication of *E. coli* infections in Buea. Susceptibility of *E. coli* to other antibiotics was lower than reported in other African countries [26,27]. *K. oxytoca* generally showed low susceptibility to antibiotics with the exception of ciprofloxacin, gentamicin and nitrofurantoin with susceptibilities more than 87% in both study sites. For *Staph spp*, ciprofloxacin (80%) was most effective against Buea isolates while for the Bamenda isolates, ampicillin (77.7%) and ceftriaxone (72.2%) were the most potent agents. We did not include a control strain in susceptibility testing. In addition, we did not determine the minimum inhibitory concentration (MIC) of potent antibiotics. These are other limitations to this study. The most inactive drugs were ampicillin, cotrimoxazole and nitrofurantoin with overall susceptibilities <50%. Higher susceptibilities to these drugs have been reported in Europe [28]. Low susceptibilities recorded in our study could be due to the fact that these antibiotics have been extensively used in the treatment of community-acquired UTIs and other infections in the past years in these regions. Most antibiotics tested are oral antibiotics which usually achieve high urinary concentrations and are therefore believed to be potent. Low susceptibilities observed indicate the need for antibiotic susceptibility testing to circumvent treatment failure. Although no reliable data exists, it is well known that many persons in study sites self-medicate, suggesting uncontrolled consumption of these antimicrobial agents particularly as they are cheap. Furthermore the implementation of policy on sale of antibiotics in study sites is weak: many unauthorized persons sell drugs and in pharmacies sale is not restricted exclusively to those with prescription. These factors could contribute to emergence of resistance. The IDSA guideline recommends a bench mark of 10–20% resistance at which first-line empiric therapy should be modified [25]. Thus, gentamicin with an overall susceptibility of 80.8% could be appropriate for empiric treatment of UTI in Buea. Most hospitals in study sites are small and usually do not admit patients who are not critically ill. Out-patients prescribed treatments with drugs such as gentamicin which is formulated as injection either return to the hospital for treatment as required or they get assistance from trained nurse in their neighborhood. None of the antibiotics tested is suitable for use in empiric eradication of UTIs in Bamenda. Thus urine cultures are necessary since treatment failure with empiric therapy is likely to occur. In addition studies with other antibiotics not included in our investigation may establish effective agent (s) against Bamenda isolates.

## Conclusion

Our findings demonstrate regional variation in UTI etiology as well as their antibiotic susceptibility emphasizing the need to establish local and national antimicrobial resistance monitoring systems in Cameroon to provide information for the development of CAUTI treatment guidelines. To the best of our knowledge, this is the first report in Cameroon that describes the etiology of community-acquired UTI and their antibiotic susceptibility in two distant localities.

## Abbreviations

IDSA: Infectious Disease Society of America; TSN: The Surveillance Network.

## Competing interests

The authors declare that they have no competing interests.

## Author’s contributions

JTKA as principal investigator conceived, designed and coordinated the study, interpreted data and initiated the writing of the manuscript. SY collected samples, isolated and characterized bacteria. NHA carried out antimicrobial susceptibility testing and interpretation of data. ENS assisted in interpretation of data and drafting the manuscript. All authors read and approved the final version of the manuscript.
